# Characterization of new bone formation in gout: a quantitative site-by-site analysis using plain radiography and computed tomography

**DOI:** 10.1186/ar3913

**Published:** 2012-07-13

**Authors:** Nicola Dalbeth, Aaron Milligan, Anthony J Doyle, Barnaby Clark, Fiona M McQueen

**Affiliations:** 1 Department of Medicine, University of Auckland, 85 Park Rd, Grafton, Auckland 1023, New Zealand; 2 Department of Radiology, Auckland District Health Board, 2 Park Road, Grafton, Auckland 1023, New Zealand; 3 Department of Anatomy with Radiology, University of Auckland, 85 Park Rd, Grafton, Auckland 1023, New Zealand; 4 Auckland Radiology Group, 2 Park Road, Grafton, Auckland 1023, New Zealand; 5 Department of Molecular Medicine, University of Auckland, 85 Park Rd, Grafton, Auckland 1023, New Zealand

## Abstract

**Introduction:**

Radiographic descriptions of gout have noted the tendency to hypertrophic bone changes. The aim of this study was to characterize the features of new bone formation (NBF) in gout, and to determine the relationship between NBF and other radiographic features of disease, particularly erosion and tophus.

**Methods:**

Paired plain radiographs (XR) and computed tomography (CT) scans of 798 individual hand and wrist joints from 20 patients with gout were analyzed. Following a structured review of a separate set of images, films were scored for the presence of the following features of NBF: spur, osteophyte, periosteal NBF, ankylosis and sclerosis. The relationship between NBF and other radiographic features was analyzed.

**Results:**

The most frequent forms of NBF were bone sclerosis and osteophyte. Spur and periosteal NBF were less common, and ankylosis was rare. On both XR and CT, joints with bone erosion were more likely to have NBF; for CT, if erosion was present, the odds ratios (OR) was 45.1 for spur, 3.3 for osteophyte, 16.6 for periosteal NBF, 26.6 for ankylosis and 32.3 for sclerosis, *P *for all < 0.01. Similarly, on CT, joints with intraosseous tophus were more likely to have NBF; if tophus was present, the OR was 48.4 for spur, 3.3 for osteophyte, 14.5 for periosteal NBF, 35.1 for ankylosis and 39.1 for sclerosis; *P *for all < 0.001.

**Conclusions:**

This detailed quantitative analysis has demonstrated that NBF occurs more frequently in joints affected by other features of gout. This work suggests a connection between bone loss, tophus, and formation of new bone during the process of joint remodelling in gout.

## Introduction

Classical radiological descriptions of gout highlight a number of characteristic features [1-4]. These include soft tissue masses representing tophi, and intra- and extra-articular erosions. In addition, descriptions have noted the tendency to hypertrophic bone changes [1-4]. Although these changes have been documented in the literature, the patterns of new bone formation (NBF) in gout have not been well characterized. Furthermore, the relationship between NBF and other radiographic features such as tophi and erosion is also unknown. Characterization of these changes may provide new insights into the mechanisms of bone remodelling in joints affected by gout.

Computed tomography (CT) provides excellent visualization and characterization of bone morphology, and also allows reliable assessment of tophus detection and size [5-7]. Through systematic analysis of bone changes and tophi, CT has the potential to provide new understanding of the mechanisms of bone remodelling in gout. The aim of this study was to define the features of NBF in joints affected by gout, and to determine the relationship between NBF and other radiographic features of disease, particularly bone erosion and tophus.

## Materials and methods

### Identification and definitions of new bone formation features

**Figure 1:**
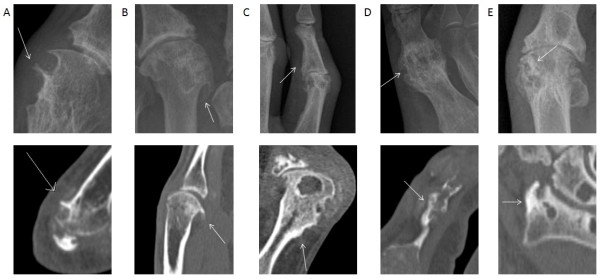
**Examples of the features of new bone formation (NBF) observed by plain radiography and computed tomography in patients with gout**. **A**. Spur. **B**. Osteophyte. **C**. Periosteal NBF. **D**. Ankylosis. **E**. Sclerosis. Top panel shows plain radiographic images and lower panel shows CT images. CT, computed tomography.

Following a review of patterns and scoring systems of NBF in other arthropathies [8-12], plain radiographs and CT scans from patients with tophaceous gout were reviewed by two rheumatologists with expertise in gout imaging (ND and FMcQ) and a musculoskeletal radiologist (AD). The review included a structured analysis of the images and identification of NBF features for assessment and measurement in the systematic site-by-site analysis. Images of the hands and the feet were used in this identification process. Definitions for all scored NBF features were agreed. A reference image library was constructed for use during the scoring process. The following NBF features were identified (with definitions): spur (a sharp spicule of dense bone proliferation extending at an acute angle from the cortex), osteophyte (bone projection arising along the joint margin and associated with cartilage [13]), periosteal NBF (bone proliferation arising from the periosteum), ankylosis (fusion of the bones of a joint, with trabeculae crossing the joint space), and sclerosis (increased density of medullary or subcortical bone). Examples of each NBF feature on plain radiography and CT are shown in Figure 1. Prior to formal scoring, both scorers (ND and AM) undertook a training calibration exercise which involved scoring five further sets of plain radiographs and CT scans.

### Site-by-site quantitative analysis of new bone formation

The site-by site analysis of NBF was completed using plain radiographs and CT scans of the hands in a further 20 patients with gout. These images were not used in the initial identification and calibration exercises described above. This dataset was selected because the clinical and radiographic features have been characterized in detail previously [6,7,14]. All patients were recruited from rheumatology clinics and had a history of acute gout according to Wallace criteria [15]. Plain radiographs and conventional CT scans of the hands were obtained for all patients. On the day of the scans, patients had a clinical assessment including gout history and laboratory testing.

Plain radiographs of the hands were obtained using a Toshiba KXO-50F X-ray machine on single-emulsion film. A standard two view examination (anteroposterior and ball-catchers views) was obtained (60 kV, 3.2 mAs). A 102 cm focal film distance with the smallest possible focal spot was used. The X-ray was centered between the second and third metacarpophalangeal (MCP) joints, perpendicular to the plane of the film. Plain radiographs were scored for erosion (0-5) and joint space narrowing (JSN) (0-4), according to the Sharp-van der Heijde (S-vdH) rheumatoid arthritis method, modified for gout [16]. This method scores erosion in the hands at the distal interphalangeal (DIP) joints, proximal interphalangeal (PIP) joints, interphalangeal (IP) joint of the thumb, MCP joints, base of the first metacarpal, multangular, scaphoid, lunate, radius and ulna.

The CT scans of the hands were performed on a Philips Brilliance 16-slice scanner (Philips Medical Systems, Best, The Netherlands), as previously described [6]. All scans were performed with the same image protocol; acquisition at 16 × 0.75 mm, reconstructed on a bone algorithm, 768 matrix, to 0.8 mm slices with a 0.4 mm increment (kVp 140, 120 mAs/slice). Additional reconstructions were done on a soft tissue algorithm, 512 matrix, also to a 0.8 mm slice with a 0.4 mm increment. The images were viewed as 0.8 mm slices on a Philips CT workstation and reconstructed to 3 mm slices for viewing on Picture Archiving Communication System (PACS). CT scans were assessed for the presence and diameter of intraosseous tophi and similarly for erosions, as previously reported [7].

The erosion and tophus scoring was undertaken more than three years before the NBF scoring. The scorers of NBF were blinded to the clinical and imaging results (including the erosion, JSN and intraosseous tophus scores). All clinical, laboratory and imaging assessments were approved by the local ethics committee, and patients provided written informed consent.

### Scoring of new bone formation

Plain radiographs and CT scans of the hands were scored for features of NBF by a rheumatologist with experience in gout imaging (ND) and a musculoskeletal radiologist (AM). Plain radiographs and CT scans were scored separately. Plain radiographs were scored first, and the scores for the plain radiographs were not available for review by the scorer at the time of the CT scoring. Sites for NBF scoring were those included in the gout-modified S-vdH method for erosion scoring (*n *= 798, two joints were not available due to finger amputation). Each NBF feature was scored as absent or present at each site by both observers. In the case of disagreement, the images were reviewed again and consensus was reached. Pre-consensus agreement for XR scoring was 93% for spur (kappa 0.58), 74% for osteophyte (kappa 0.29), 93% for periosteal NBF (kappa 0.15), 99% for ankylosis (kappa 0.40) and 86% for sclerosis (kappa 0.64). Pre-consensus agreement for CT scoring was 87% for spur (kappa 0.46), 76% for osteophyte (kappa 0.32), 94% for periosteal NBF (kappa 0.15), 99% for ankylosis (kappa 0.33) and 86% for sclerosis (kappa 0.69). In addition, if a spur was present on CT, the spur length was measured in millimetres (mm) as the distance from the point at which cortex changes angle to the tip of spur. In the presence of sclerosis, the relative density was assessed by measuring the Hounsfield units (HU) at the site of sclerosis, and at adjacent cortical and trabecular bone, using the CT workstation software. For spur length the intraclass correlation coefficient was 0.71 (95% confidence interval (95% CI) 0.67 to 0.74) and for sclerosis HU was 0.64 (95% CI 0.60 to 0.68).

### Statistical analysis

Data were analysed using Prism (v5, GraphPad, San Diego, CA) and SPPS (v15, SPSS Inc., Chicago, IL). Mean with standard deviations (SD) and percentages were used to describe NBF scores. For the site analysis, joints were grouped into the DIP joints, PIP joints (including the IP joint of the thumb), MCP joints, carpal region (base of the first metacarpal, multangular, scaphoid, lunate), and radius/ulna. To account for the different number of joints at each site, data were expressed as the percentage affected/patient. HU at different bone sites and NBF features at the different joint sites were analyzed by repeated measures analysis of variance (ANOVA) with Tukey's multiple comparison *post hoc *test. Radiographic and CT NBF data were analyzed using unpaired t tests and Fisher's exact tests. For the purposes of the site-by-site analysis, each joint was considered an independent unit for analysis. To address the possibility that lesions were nested within individuals, the number of affected joints per patient was also used in Pearson correlation analysis. All tests were two tailed and *P *< 0.05 was considered significant.

## Results

### Patient characteristics

The clinical and imaging characteristics of the patients have been reported in detail previously [6,7,14]. There were 19 (95%) men with mean (SD) age of 56.0 (10.8) years. The mean (SD) disease duration was 19.5 (12.8) years and serum urate was 0.49 (0.13) mmol/L. Eleven (55%) has microscopically proven disease. The majority of patients (16/20, 80%) had subcutaneous tophi, and the mean (SD) number of subcutaneous tophi was 30 (56). On plain radiographs of the hands, erosion was present in 270/798 (33.8%) joints and joint space narrowing in 113/558 (20.3%) scored sites. On plain radiographs of the hands, the mean (SD) modified S-vdH erosion score was 37 (39), narrowing score was 17 (22) and combined score was 54 (59). On CT scanning of the hands, there were 237/798 (29.7%) joints with erosion and 194/798 (24.3%) joints with intraosseous tophus. For those joints with bone erosion, the mean (SD) erosion diameter was 4.8 (3.9) mm. For those joints with intraosseous tophus, the mean (SD) tophus diameter was 9.6 (7.3) mm.

### Features of new bone formation on plain radiographs and CT scans

**Figure 2:**
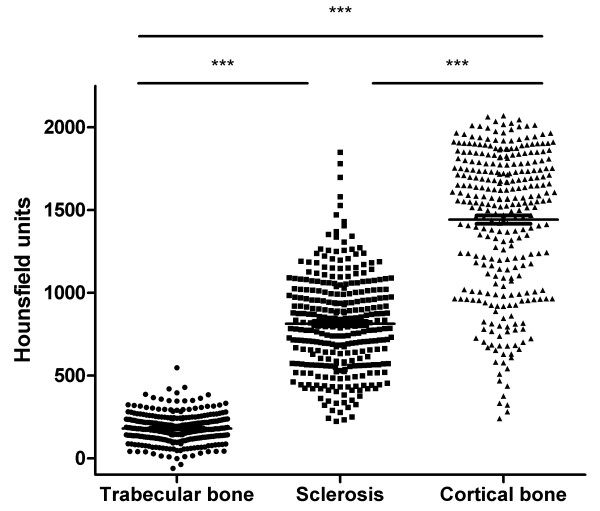
**Hounsfield units at sites of sclerosis and adjacent bone in joints affected by bone sclerosis**. ***P < 0.0001 by Tukey's *post hoc *test.

The features of NBF observed in the hand radiographs and CT scans are shown in Table 1. The most frequent forms of NBF were bone sclerosis and osteophyte. Spur and periosteal NBF were less common, and ankylosis was rare. CT detected more spurs and osteophytes than plain radiography (Table 1). Agreement between plain radiographs and CT for the features of NBF was 88% for spur (kappa 0.51), 81% for osteophyte (kappa 0.52), 96% for periosteal NBF (kappa 0.37), 99% for ankylosis (kappa 0.54) and 90% for sclerosis (kappa 0.75). For those spurs identified on CT, the mean (SD) spur length was 4.4 (2.3) mm. For those sites of bone sclerosis identified on CT, the relative density of sclerosis was intermediate between trabecular and cortical bone; mean (SD) HU for sclerosis was 814 (288), compared with 180 (86) for adjacent trabecular bone and 1,443 (431) for cortical bone, ANOVA *P *< 0.0001 (Figure 2). These results confirmed the increased density of trabecular bone at sites of bone sclerosis.

**Table 1:** Features of new bone formation (NBF) in patients with gout.

	Number of joints with XR NBF features (number = 798 joints)	Mean (SD) number of joints with XR NBF features (number = 20 patients)	Number of joints with CT NBF features (number = 798 joints)	Mean (SD) number of joints with CT NBF features (number = 20 patients)
Spur	76 (9.5%)	3.8 (5.7)	141 (17.6%)	7.1 (7.9)
Osteophyte	167 (20.9%)	8.4 (6.5)	243 (30.5%)	12.2 (7.7)
Periosteal NBF	51 (6.4%)	2.6 (3.4)	48 (6.0%)	2.4 (3.4)
Ankylosis	7 (0.9%)	0.35 (1.1)	5 (0.6%)	0.25 (3.4)
Sclerosis	207 (25.9%)	10.4 (8.9)	228 (28.6%)	11.4 (8.3)

CT, computed tomography; SD, standard deviation; XR, plain radiography.

### Relationship of new bone formation with other radiographic features on plain radiography

Site-by-site analysis of the plain radiographs showed that NBF was strongly associated with bone erosion and joint space narrowing (Table 2). Those joints with bone erosion were more likely to be affected by all features of NBF compared with non-eroded joints. Furthermore, erosion scores were higher in eroded joints that were affected by NBF. Similarly, joints with joint space narrowing were more likely to be affected by NBF, with higher narrowing scores in narrowed joints affected by spur, ankylosis and sclerosis. Overall, the relationship of osteophyte with erosion and joint space narrowing was weaker than other forms of NBF.

**Table 2:** Site-by-site plain radiography analysis.

		Erosion present, number	Erosion absent, number	OR (95%CI)	*P*	Erosion score, in eroded joints, mean (SD)	P	JSN present, number	JSN absent, number	OR (95%CI)	*P*	JSN score, in narrowed joints, mean (SD)	*P*
Spur	Present	73	3	64.9	<0.0001	4.2 (1.2)	<0.0001	55	14	28.7	<0.0001	2.6 (1.2)	<0.0001
	Absent	197	525	(20.2-208.5)		2.2 (1.3)		58	431	(15.0-54.8)		1.8 (0.9)	
Osteophyte	Present	139	28	19.0	<0.0001	3.2 (1.5)	<0.0001	75	58	13.2	<0.0001	2.2 (1.1)	0.65
	Absent	131	500	(12.1-29.7)		2.3 (1.4)		38	387	(8.2-21.2)		2.3 (1.2)	
Periosteal NBF	Present	50	1	119.8	<0.0001	3.9 (1.4)	<0.0001	29	7	21.6	<0.0001	2.5 (1.1)	0.08
	Absent	220	527	(16.4-872.9)		2.5 (1.5)		84	438	(9.2-51.0)		2.1 (1.1)	
Ankylosis	Present	7	0	30.1	0.0005	5.0 (0.0)	<0.0001	5	0	45.2	0.0003	4.0 (0.0)	<0.0001
	Absent	263	528	(1.7-529.)		2.7 (1.5)		108	445	(2.5-823.6)		2.1 (1.1)	
Sclerosis	Present	194	13	101.1	<0.0001	3.2 (1.5)	<0.0001	91	62	25.6	<0.0001	2.4 (1.1)	<0.0001
	Absent	76	515	(54.9-186.3)		1.5 (0.7)		22	383	(14.9-43.7)		1.4 (0.7)	

Relationship of new bone formation (NBF) with erosion and joint space narrowing (JSN) (*n *= 798 joints for erosion and 558 joints for JSN). CI, confidence interval; OR, odds ratio; SD, standard deviation.

### Relationship of new bone formation with erosion and tophus on computed tomography: site-by-site analysis

Site-by-site analysis of the CT scans also showed that joints with bone erosion were more likely to have NBF than those without erosion (Table 3). On CT scanning, erosions were larger in eroded joints that were affected by certain forms of NBF: spur, periosteal NBF and sclerosis. Similarly, joints with intraosseous tophus were more likely to have NBF than those without tophus, although the relationship between intraosseous tophus size and NBF was weaker (Table 3). Spur length correlated with both erosion diameter r = 0.67 (0.63 to 0.71) *P *< 0.0001 and tophus diameter r = 0.67 (0.63 to 0.71) *P *< 0.0001. Tophus density, measured in HU, was not associated with features of NBF (data not shown).

**Table 3:** Site-by-site CT analysis.

		Erosion present, number	Erosion absent, number	OR (95%CI)	*P*	Erosion diameter, in eroded joints, mean (SD)	*P*	Tophus present, number	Tophus absent, number	OR (95%CI)	*P*	Tophus diameter, in joints with IO tophus, mean (SD)	*P*
Spur	Present	127	14	45.1	<0.0001	7.0 (3.7)	<0.0001	121	20	48.4	<0.0001	10.9 (7.9)	0.02
	Absent	110	547	(25.0-81.3)		4.5 (3.0)		73	584	(28.4-82.4)		8.2 (6.7)	
Osteophyte	Present	116	127	3.3	<0.0001	5.6 (3.2)	0.27	99	144	3.3	<0.0001	8.4 (5.3)	0.006
	Absent	121	434	(2.4-4.5)		6.1 (3.9)		95	460	(2.4-4.7)		11.3 (9.1)	
Periosteal NBF	Present	41	7	16.6	<0.0001	7.2 (4.4)	0.0005	38	10	14.5	<0.0001	10.4 (7.9)	0.65
	Absent	196	554	(7.3-37.5)		5.5 (3.3)		156	594	(7.1-27.0)		9.7 (7.5)	
Ankylosis	Present	5	0	26.6	0.002	8.3 (3.1)	0.11	5	0	35.1	0.0008	10.7 (7.3)	0.80
	Absent	232	561	(1.5-482.7)		5.8 (3.8)		189	604	(1.9-638.0)		9.8 (7.6)	
Sclerosis	Present	179	49	32.3	<0.0001	6.5 (3.8)	<0.0001	161	67	39.1	<0.0001	10.4 (7.9)	0.03
	Absent	58	512	(21.3-48.9)		3.9 (2.0)		33	537	(24.9-61.5)		7.3 (4.6)	

Relationship of new bone formation (NBF) with erosion and intraosseous tophus on CT (*n *= 798 joints). CI, confidence interval; CT, computed tomography; OR, odds ratio; SD, standard deviation.

### Relationship of new bone formation with erosion and tophus on computed tomography: patient level analysis

In the patient level analysis, the number of joints affected by NBF on CT scanning did not correlate with gout disease duration or number of attacks in the preceding six months (data not shown). Serum urate correlated with the number of joints affected by bone spur (r = 0.54, *P *= 0.01) and ankylosis (r = 0.54, *P *= 0.01) on CT, but not other features of NBF. Similarly, the number of subcutaneous tophi correlated with the number of joints affected by bone spur (r = 0.50, *P *= 0.02) and ankylosis (r = 0.66, *P *= 0.001) on CT, but not other features of NBF. Consistent with the site-by-site analysis, the number of joints affected by CT erosion and intraosseous tophus correlated with the number of joints affected by bone spur, sclerosis, ankylosis and periosteal NBF on CT scanning (Table 4). In contrast, there was no relationship between the number of joints affected by CT erosion and intraosseous tophus and the number of joints affected by osteophyte.

**Table 4:** Patient level CT analysis.

	Spur	Osteophyte	Periosteal NBF	Ankylosis	Sclerosis
Number of joints with CT erosion	0.88***	0.38	0.60**	0.67**	0.90***
Number of joints with CT intraosseous tophus	0.87***	0.31	0.56*	0.70***	0.82***

Spearman correlations between number of joints affected by CT new bone formation (NBF) and other imaging features of gout (*n *= 20 patients). * < 0.05, ** < 0.01, *** < 0.001. CT, computed tomography; n, number.

Further analysis was undertaken by examining CT involvement within groups of joints (Table 5). Erosion was more commonly observed at the radius/ulna site than at the DIP joints (repeated measures one way ANOVA *P *= 0.03, Tukey post hoc test *P *< 0.05), with no other differences between other sites. Similarly tophi were more frequently observed in the PIP joints and radius/ulna site compared with the DIP joints (repeated measures one way ANOVA *P *= 0.01, Tukey post hoc test *P *< 0.05 for both). There was no difference between sites for spurs, osteophytes or ankylosis. The radius/ulna site was more frequently affected by sclerosis and periosteal new bone formation compared with other sites (Table 5).

**Table 5:** Sites of NBF and other features of gout on CT.

	Distal interphalangeal joints	Proximal interphalangeal joints^a^	Metacarpophalangeal joints	Carpal region	Radius and ulna
Spur	12.5 (21.1)	20.0 (22.7)	18.0 (25.5)	16.3 (26.6)	24.8 (32.9)
Osteophyte	23.1 (25.1)	41.0 (23.2)	24.5 (24.2)	33.1 (32.3)	27.5 (30.2)
Periosteal NBF	1.9 (6.1)***	6.0 (10.5)*	2.5 (5.5)**	9.4 (16.1)	16.3 (23.3)
Ankylosis	0.0 (0.03)	1.5 (3.7)	0.0 (0.0)	0.6 (2.8)	0.0 (0.0)
Sclerosis	18.1 (23.5)**	35.0 (25.2)	23.5 (25.8)*	30.6 (28.8)	41.3 (33.7)
Erosion	20.9 (22.6)*	35.8 (26.4)	26.5 (29.8)	29.4 (31.5)	41.3 (40.8)
Tophus	21.4 (26.7)*	39.3 (31.2)	34.0 (36.6)	29.4 (32.5)	41.3 (43.9)

^a^Includes IP joint of the thumb. *P < 0.05 compared with radius/ulna, **P < 0.01 compared with radius/ulna, ***P < 0.001 compared with radius/ulna. All *P *refer to Tukey's *post hoc *test. Data are expressed as a mean (standard deviation) percentage of sites per patient to account for the different number of joints at each site. CT, computed tomography; NBF, new bone formation.

In the patient level analysis, the percentage of joints affected by spur and sclerosis correlated highly with the percentage of joints affected by tophus and erosion at each group of joints (r = 0.62-0.97, *P *< 0.01 for all). With the exception of tophus at the PIP joints, the percentage of joints affected by periosteal NBF also correlated with the percentage of joints affected by tophus and erosion at each site (r = 0.50-0.88, *P *< 0.05 for all). At the DIP joints and the PIP joints, there was no correlation between the percentage of joints affected by osteophytes and the percentage of joints affected by erosion or tophus (*P *> 0.52 for all). However, at the other joints sites (MCP joints, wrists and radius/ulna site), there was a modest correlation between percentage of joints affected by osteophytes and the percentage of joints affected by erosion (r = 0.47, *P *< 0.05 for all).

## Discussion

This detailed quantitative analysis has demonstrated that NBF is closely related to other features of joint disease in gout. The most common features of NBF in gout are sclerosis, osteophyte and spur. The finding that bone erosion is strongly associated with all features of NBF suggests that loss of bone and formation of new bone may be connected during the joint remodelling process in joints affected by gout.

Although tophus size was not strongly associated with features of NBF in this analysis, joints with intraosseous tophus were more likely to have associated NBF, compared with those joints without tophus. These data suggest that the tophus may play a role in development of these features, particularly spur, periosteal NBF, ankylosis and sclerosis. The tophus represents a foreign body granulomatous response to collections of monosodium urate crystals, involving innate and adaptive immune cells [17]. It seems unlikely that MSU crystals within tophi have a direct effect on bone cells to promote NBF, as these crystals promote osteoclastogenesis and inhibit osteoblast differentiation, survival and function, resulting in net bone resorption [18-21]. MSU crystals within the tophus are surrounded by an inflammatory cell rim (the corona zone) which, in turn, is surrounded by a fibrovascular zone with organized deposition of collagen [22]. It is conceivable that the processes that contribute to formation of the fibrovascular zone also contribute to the development of some forms of NBF, such as bone sclerosis. In addition to expression of cytokines typically associated with bone resorption, such as IL-1 and TNF-α [17,23], transforming growth factor β (TGFβ) is also expressed in the tophus [17]. TGFβ has various effects on bone homeostasis, but may contribute to NBF under certain conditions [24]. Other pathways implicated in NBF in other arthropathies such as the bone morphogenetic proteins (BMPs) and Wnt signalling pathways have not been explored to date in patients with gout; activation of these pathways may also contribute to the patterns of NBF in joints affected by gout [25-27].

Magnetic resonance imaging studies have demonstrated a close relationship between resolution of inflammatory bone lesions and development of new bone formation in ankylosing spondylitis [28,29]. Our study was not able to address bone inflammation at sites of NBF, as this feature is not visualized using CT. The current study has defined the features of NBF that occur in patients with gout. Longitudinal multimodality imaging studies are now needed to understand the mechanisms of NBF in gout, and the impact of treatment on these features.

It is also possible that elevated circulating concentrations of soluble urate contribute to NBF in patients with gout. Several studies have reported that serum urate concentrations positively correlate with total body bone mineral density [30,31]. A large observational study of older men has demonstrated that higher serum urate concentrations are associated with lower prevalence of osteoporosis and osteoporotic fractures [31]. These findings persisted after adjusting for potential confounders including body mass index, alcohol use, diuretic therapy and kidney disease. The mechanisms of this relationship are not fully understood, but analysis of bone turnover markers showed that serum urate negatively correlated with urinary NTX-1, a marker of bone resorption [31]. In contrast, there was no relationship observed between serum urate concentrations and P1NP, a marker of osteoblast activity. These observations suggest that urate may have opposing effects on bone, leading to erosion at local sites in the context of MSU crystals in tophi, but also maintenance of total bone density in response to high soluble urate concentrations. It is unknown whether the mechanisms of local NBF within joints affected by MSU crystals are the same as those influencing total body bone density. In this analysis, we did identify a weak relationship between serum urate and the number of joints affected by bone spur and ankylosis, but not other forms of NBF.

It is well documented that gout is more likely to present in joints previously affected by osteoarthritis [32]. Consistent with these observations, osteophytes were associated with the presence of joint space narrowing, erosion and intraosseous tophus in the site-by-site analysis. However, the relationship of erosion and intraosseous tophus with osteophyte was much weaker than with the other forms of NBF. Furthermore, in the overall patient level analysis, a relationship between osteophyte and NBF was not observed. The analysis of different joint areas showing a relationship between erosion and tophus with osteophytes at sites other than the DIP and PIP joints suggests that these processes may be related in some circumstances and not in others.

This study shows that erosion, tophus and certain features of NBF (periosteal new bone formation and sclerosis) are more common at the radius/ulna region compared with the distal interphalangeal joints. It is possible this difference may have been due to improved resolution at the larger site, compared with the small DIP joints. However, it is more likely that this represents a real difference. Potential explanations for this observation are that local factors such as biomechanical strain at the distal radioulnar region contribute to formation of MSU crystals, or promotion of tophus formation in the presence of MSU crystals at this site.

Patients in this study had longstanding gout with a mean disease duration of almost 20 years and high serum urate levels. Although we did not observe a relationship between disease duration and NBF features in the patient level analysis, the results described in this paper may not necessarily be applied to patients with early disease or with well controlled disease. Similarly, this study only included joints of the hands, and it is possible that NBF changes may occur at different rates in the feet, which are more frequently affected by gout. Analysis of the feet is planned in future studies.

A further question that arises from this work is whether CT is preferable to plain radiography to accurately detect features of NBF. CT was able to detect more osteophytes and spurs than plain radiography. However, the inter-reader agreement was similar between XR and CT, with reasonable agreement between the methods for detection of the various features of NBF, suggesting that plain radiographs may be sufficient. The additional cost and radiation exposure from CT further supports plain radiography as the method of choice.

The strong relationship between bone loss (erosion) and NBF observed in this cross-sectional study suggests that NBF features such as sclerosis and spur formation may be repair phenomena triggered by joint destruction. At present, it is unclear whether NBF in gout occurs before bone erosion and tophus formation, develops concurrently with bone erosion, or occurs as part of tissue remodelling in response to tophus and erosion. Exploration of the temporal relationship between tophus, NBF and bone erosion now requires detailed longitudinal studies. Ideally these will be multimodality imaging studies which include plain radiography, MRI and dual energy CT, an advanced imaging method that allows visualization of urate deposits [33]. Intervention studies are also needed to address whether effective urate-lowering therapy can prevent or reverse features of NBF, and to understand the impact of these changes on musculoskeletal function. Evaluation of NBF as part of the imaging assessment in future studies of gout may clarify the pathogenesis and impact of NBF in this disease.

## Conclusions

This detailed quantitative analysis has demonstrated that NBF occurs more frequently in joints affected by other features of gout. This work suggests a connection between bone loss, tophus, and formation of new bone during the process of joint remodelling in gout.

## Abbreviations

ANOVA: analysis of variance; CI: confidence interval; CT: computed tomography; DIP: distal interphalangeal; HU: Hounsfield units; IL: interleukin; IP: interphalangeal; JSN: joint space narrowing; MCP: metacarpophalangeal; NBF: new bone formation; OR: odds ratio; PIP: proximal interphalangeal; SD: standard deviation; S-vdH: Sharp-van der Heijde; TGF: transforming growth factor; TNF: tumour necrosis factor; XR: plain radiography.

## Competing interests

The authors declare that they have no relevant conflicts of interest.

## Authors' contributions

ND (the guarantor) accepts full responsibility for the work and the conduct of the study, had access to the data, and controlled the decision to publish. ND conceived of the study, and contributed to the data acquisition, analysis and interpretation. AD and FM contributed to the design of the study protocol, data acquisition, and interpretation of the data. AM and BC contributed to the data acquisition and interpretation. All authors read and approved the final manuscript.

## References

[B1] Resnick D Broderick TW Intraosseous calcifications in tophaceous gout AJR Am J Roentgenol 1981 137 1157 1161 6976085 10.2214/ajr.137.6.1157

[B2] Watt I Middlemiss H The radiology of gout. Review article Clin Radiol 1975 26 27 36 804372 10.1016/s0009-9260(75)80004-3

[B3] Barthelemy CR Nakayama DA Carrera GF Lightfoot RW Jr Wortmann RL Gouty arthritis: a prospective radiographic evaluation of sixty patients Skeletal Radiol 1984 11 1 8 10.1007/BF00361124 6710175

[B4] Bloch C Hermann G Yu TF A radiologic reevaluation of gout: a study of 2,000 patients AJR Am J Roentgenol 1980 134 781 787 6767366 10.2214/ajr.134.4.781

[B5] Gerster JC Landry M Duvoisin B Rappoport G Computed tomography of the knee joint as an indicator of intraarticular tophi in gout Arthritis Rheum 1996 39 1406 1409 10.1002/art.1780390820 8702451

[B6] Dalbeth N Clark B Gregory K Gamble GD Doyle A McQueen FM Computed tomography measurement of tophus volume: comparison with physical measurement Arthritis Rheum 2007 57 461 465 10.1002/art.22612 17394233

[B7] Dalbeth N Clark B Gregory K Gamble G Sheehan T Doyle A McQueen FM Mechanisms of bone erosion in gout: a quantitative analysis using plain radiography and computed tomography Ann Rheum Dis 2009 68 1290 1295 10.1136/ard.2008.094201 18708415

[B8] Rahman P Gladman DD Cook RJ Zhou Y Young G Salonen D Radiological assessment in psoriatic arthritis Br J Rheumatol 1998 37 760 765 10.1093/rheumatology/37.7.760 9714353

[B9] Taylor WJ Porter GG Helliwell PS Operational definitions and observer reliability of the plain radiographic features of psoriatic arthritis J Rheumatol 2003 30 2645 2658 14719209

[B10] Ostergaard M McQueen F Wiell C Bird P Boyesen P Ejbjerg B Peterfy C Gandjbakhch F Duer-Jensen A Coates L Haavardsholm EA Hermann KG Lassere M O'Connor P Emery P Genant H Conaghan PG The OMERACT psoriatic arthritis magnetic resonance imaging scoring system (PsAMRIS): definitions of key pathologies, suggested MRI sequences, and preliminary scoring system for PsA Hands J Rheumatol 2009 36 1816 1824 10.3899/jrheum.090352 19671819

[B11] Wendling D Toussirot E Streit G Prati C Imaging study scores for ankylosing spondylitis Joint Bone Spine 2006 73 655 660 10.1016/j.jbspin.2006.03.007 17064946

[B12] Buckland-Wright C Subchondral bone changes in hand and knee osteoarthritis detected by radiography Osteoarthritis Cartilage 2004 12 Suppl A S10 19 14698636 10.1016/j.joca.2003.09.007

[B13] Resnick D Diagnosis of Bone and Joint Disorders 2002 4 Philadelphia: Saunders

[B14] Dalbeth N Collis J Gregory K Clark B Robinson E McQueen FM Tophaceous joint disease strongly predicts hand function in patients with gout Rheumatology (Oxford) 2007 46 1804 1807 10.1093/rheumatology/kem246 17982165

[B15] Wallace SL Robinson H Masi AT Decker JL McCarty DJ Yu TF Preliminary criteria for the classification of the acute arthritis of primary gout Arthritis Rheum 1977 20 895 900 10.1002/art.1780200320 856219

[B16] Dalbeth N Clark B McQueen F Doyle A Taylor W Validation of a radiographic damage index in chronic gout Arthritis Rheum 2007 57 1067 1073 10.1002/art.22891 17665492

[B17] Dalbeth N Pool B Gamble GD Smith T Callon KE McQueen FM Cornish J Cellular characterization of the gouty tophus: a quantitative analysis Arthritis Rheum 2010 62 1549 1556 10.1002/art.27356 20131281

[B18] Lee SJ Nam KI Jin HM Cho YN Lee SE Kim TJ Lee SS Kee SJ Lee KB Kim N Park YW Bone destruction by receptor activator of nuclear factor kappaB ligand-expressing T cells in chronic gouty arthritis Arthritis Res Ther 2011 13 R164 10.1186/ar3483 21992185 PMC3308097

[B19] Dalbeth N Smith T Nicolson B Clark B Callon K Naot D Haskard DO McQueen FM Reid IR Cornish J Enhanced osteoclastogenesis in patients with tophaceous gout: urate crystals promote osteoclast development through interactions with stromal cells Arthritis Rheum 2008 58 1854 1865 10.1002/art.23488 18512794

[B20] Bouchard L de Medicis R Lussier A Naccache PH Poubelle PE Inflammatory microcrystals alter the functional phenotype of human osteoblast-like cells in vitro: synergism with IL-1 to overexpress cyclooxygenase-2 J Immunol 2002 168 5310 5317 11994489 10.4049/jimmunol.168.10.5310

[B21] Chhana A Callon KE Pool B Naot D Watson M Gamble GD McQueen FM Cornish J Dalbeth N Monosodium urate monohydrate crystals inhibit osteoblast viability and function: implications for development of bone erosion in gout Ann Rheum Dis 2011 70 1684 1691 10.1136/ard.2010.144774 21622970

[B22] Palmer DG Highton J Hessian PA Development of the gout tophus. An hypothesis Am J Clin Pathol 1989 91 190 195 2916461 10.1093/ajcp/91.2.190

[B23] Schweyer S Hemmerlein B Radzun HJ Fayyazi A Continuous recruitment, co-expression of tumour necrosis factor-alpha and matrix metalloproteinases, and apoptosis of macrophages in gout tophi Virchows Arch 2000 437 534 539 10.1007/s004280000282 11147175

[B24] Joyce ME Roberts AB Sporn MB Bolander ME Transforming growth factor-beta and the initiation of chondrogenesis and osteogenesis in the rat femur J Cell Biol 1990 110 2195 2207 10.1083/jcb.110.6.2195 2351696 PMC2116133

[B25] Fujimori Y Nakamura T Ijiri S Shimizu K Yamamuro T Heterotopic bone formation induced by bone morphogenetic protein in mice with collagen-induced arthritis Biochem Biophys Res Commun 1992 186 1362 1367 10.1016/S0006-291X(05)81556-6 1510667

[B26] Lories RJ Derese I Luyten FP Modulation of bone morphogenetic protein signaling inhibits the onset and progression of ankylosing enthesitis J Clin Invest 2005 115 1571 1579 10.1172/JCI23738 15902307 PMC1090472

[B27] Diarra D Stolina M Polzer K Zwerina J Ominsky MS Dwyer D Korb A Smolen J Hoffmann M Scheinecker C van der Heide D Landewe R Lacey D Richards WG Schett G Dickkopf-1 is a master regulator of joint remodeling Nat Med 2007 13 156 163 10.1038/nm1538 17237793

[B28] Pedersen SJ Chiowchanwisawakit P Lambert RG Ostergaard M Maksymowych WP Resolution of inflammation following treatment of ankylosing spondylitis is associated with new bone formation J Rheumatol 2011 38 1349 1354 10.3899/jrheum.100925 21459937

[B29] Maksymowych WP Chiowchanwisawakit P Clare T Pedersen SJ Ostergaard M Lambert RG Inflammatory lesions of the spine on magnetic resonance imaging predict the development of new syndesmophytes in ankylosing spondylitis: evidence of a relationship between inflammation and new bone formation Arthritis Rheum 2009 60 93 102 10.1002/art.24132 19116919

[B30] Dalbeth N Horne A Gamble GD Ames R Mason B McQueen FM Bolland MJ Grey A Reid IR The effect of calcium supplementation on serum urate: analysis of a randomized controlled trial Rheumatology (Oxford) 2009 48 195 197 19036779 10.1093/rheumatology/ken416

[B31] Nabipour I Sambrook PN Blyth FM Janu MR Waite LM Naganathan V Handelsman DJ Le Couteur DG Cumming RG Seibel MJ Serum uric acid is associated with bone health in older men: a cross-sectional population-based study J Bone Miner Res 2011 26 955 964 10.1002/jbmr.286 21541998

[B32] Roddy E Zhang W Doherty M Are joints affected by gout also affected by osteoarthritis? Ann Rheum Dis 2007 66 1374 1377 10.1136/ard.2006.063768 17284542 PMC1994292

[B33] Choi HK Al-Arfaj AM Eftekhari A Munk PL Shojania K Reid G Nicolaou S Dual energy computed tomography in tophaceous gout Ann Rheum Dis 2009 68 1609 1612 10.1136/ard.2008.099713 19066180

